# The Number of Myomas Is the Most Important Risk Factor for Blood Loss and Total Operation Time in Robotic Myomectomy: Analysis of 242 Cases

**DOI:** 10.3390/jcm10132930

**Published:** 2021-06-30

**Authors:** Sa Ra Lee, Ju Hee Kim, Sehee Kim, Sung Hoon Kim, Hee Dong Chae

**Affiliations:** 1 Asan Medical Center, Department of Obstetrics and Gynecology, University of Ulsan College of Medicine, 88, Olympic-ro 43-gil, Seoul 05505, Korea; xjuheex@gmail.com (J.H.K.); kimsung@amc.seoul.kr (S.H.K.); hdchae@amc.seoul.kr (H.D.C.); 2 Asan Medical Center, Department of Clinical Epidemiology and Biostatistics, University of Ulsan College of Medicine, 88, Olympic-ro 43-gil, Seoul 05505, Korea; seheek2050@gmail.com

**Keywords:** blood loss, complication, myoma, operation time, robotic myomectomy

## Abstract

To identify factors affecting blood loss and operation time (OT) during robotic myomectomy (RM), we reviewed a total of 448 patients who underwent RM at Seoul Asan Hospital between 1 January 2019, and 28 February 2021, at Seoul Asan Hospital. To avoid variations in surgical proficiency, only 242 patients managed by two surgeons who each performed >80 RM procedures during the study period were included in this study. All cases of RM were performed with a reduced port technique. We obtained the following data from each patient’s medical chart: age, gravidity, parity, body mass index, and history of previous abdominal surgery including cesarean section. We also collected information on the maximal diameter and type of myomas, number and weight of removed myomas, concomitant surgery, total OT from skin incision to closure, estimated blood loss (EBL), and blood transfusion. Data on preoperative use of gonadotropin-releasing hormone agonists (GnRHas) and perioperative use of hemostatic agents (tranexamic acid or vasopressin) were also collected. Data on the length of hospital stay, postoperative fever within 48 h, and any complications related to RM were also obtained. The primary endpoint in this study was the identification of factors affecting EBL and the secondary endpoint was the identification of factors affecting the total OT during multiport RM. Univariate and multivariate analyses were used to identify the factors affecting EBL and OT during multiport RM. The medians of the maximal diameter and weight of the removed myomas were 9.00 (interquartile range [IQR], 7.00 to 10.00) cm and 249.75 (IQR, 142.88 to 401.00) g, respectively. The median number of myomas was two (IQR, one to four), ranging from 1 to 34. Of the cases, 155 had low EBL and 87 had high EBL. Most myomas were of the intramural type (n = 179). The odds of EBL > 320 mL increased by 251% (odds ratio [OR], 2.51; 95% confidence interval [CI], 1.16–5.42) for five to nine myomas and by 647% (OR, 6.47; 95% CI, 1.87–22.33) for ≥10 myomas. The odds of subserosal-type myomas decreased by 67% compared with intramural-type myomas (OR, 0.33; 95% CI, 0.14–0.80). History of abdominal surgery other than cesarean section was positively correlated with EBL. The weight of the removed myomas and a history of previous cesarean section were not correlated with the EBL. *Conclusion*: The number of myomas (5–9 and ≥10), maximal myoma diameter, and history of abdominal surgery other than cesarean section affect the EBL in RM.

## 1. Introduction

Uterine myoma is a common benign gynecologic tumor in reproductive-aged women, and myomectomy, the standard fertility-preserving surgical option, is increasingly being performed [1,2]. Women who underwent myomectomy had substantial improvement in health-related quality of life, regardless of the route of myomectomy, even in women older than age 40 [3,4]. A minimally invasive approach is preferable to an open approach, particularly with respect to better perioperative outcome considering that patients who underwent open myomectomy took two weeks longer to return to work than those who underwent laparoscopic myomectomy (LM) [3]. In addition to that, minimally invasive myomectomies decrease the postoperative adhesion formation, which are important to optimize the reproductive outcomes of patients [5,6]. LM can be conducted with a low rate of major complications including uterine rupture during pregnancy [7,8] and in terms of estimated blood loss (EBL) during myomectomy, surgical approaches such as laparotomy, conventional laparoscopy, or robot-assisted laparoscopy can have an effect, in terms of not only the length of skin incision but also the available methods for hemostasis including manual compression (available only in laparotomy) and the ease of rapid myometrial suturing.

Among the procedures in myomectomy, fast and multilayer myometrial suturing after myoma retrieval is crucial not only for minimizing the risk of uterine rupture during pregnancy but also for providing effective hemostasis and decreasing the EBL [9,10].

RM has many advantages, such as three-dimensional high-definition magnification, a stable ergonomic platform, elimination of physiologic tremors, and motion scaling [11,12,13,14]; however, higher EBL and longer operation time (OT) than in LM were often reported as disadvantages of RM, especially in the early period of the use of this technique [13,15]. Although a recent meta-analysis including a total of 2027 patients found no significant difference between RM and LM in terms of OT, blood loss, transfusion, length of hospital stay, complications, or postoperative fertility [16], RM may have some disadvantages in terms of the limited available hemostasis, surgical techniques, and instruments.

High EBL increases the risk of transfusion, acute renal insufficiency, readmission, and reoperation [17,18,19,20], and long OT increases perioperative complications including decreased body temperature, longer hospital stays, and longer recovery [21]. Therefore, surgeons make efforts to decrease the EBL and total OT to minimize operation-related complications. The preoperative prediction of EBL and total OT is always important for safe surgery, especially in myomectomy because this procedure is known to have higher EBL than other surgical options for symptomatic uterine myomas, including total hysterectomy and supracervical hysterectomy [18,22].

Therefore, this study aimed to identify preoperative risk factors independently associated with a higher EBL and longer OT as potential adverse surgical outcomes of multiport RM. The primary endpoint was to identify factors affecting EBL to effectively prepare for high EBL by evaluating the risk factors for high EBL. The secondary endpoint was to identify factors affecting the total OT during multiport RM.

## 2. Materials and Methods

### 2.1. Definitions of Primary and Secondary Outcomes

The primary outcome was defined as high EBL (>320 mL) or low EBL (≤320 mL), and the secondary outcome was defined as a total OT > 3 or ≤3 h.

For the purpose of this study, we calculated the EBL as follows: intraperitoneal bleeding was suctioned through surgical drains into a suction bottle, which can be used to determine the volume of fluids. To determine an accurate EBL, irrigation with normal saline was minimized during surgery. Intraperitoneal blood suction was performed during and after the end of the procedure. Then, the EBL was recorded immediately. Entire intraperitoneal irrigation with normal saline was performed before abdominal closure; this was not included in the EBL. In addition, two experienced surgeons in this study reviewed the EBL to minimize bias.

Since the distribution of the EBL on a continuous scale was heavily right-skewed, patients were classified into two groups: high EBL (>320 mL) and low EBL (≤320 mL), and these were used as the primary outcome variables. The threshold for a high vs. low EBL value was determined based a median EBL of 300 mL (IQR, 200–400 mL) and a typical unit of packed red cells of 320 mL/pint. Clinical relevance with similar thresholds has been shown in several previous surgical studies [21,22].

Similarly, based on the right-skewed distribution of the total OT, the secondary outcome was defined as a binary variable: total OT > 3 h vs. ≤3 h. The median OT of our data was 144 min with an IQR of 112 to 182 min, and the threshold for a longer OT was determined based on the upper quartile (top 25%) of OT values considering clinical relevance. 

However, we prefer not to conduct any post hoc power analysis for this retrospective study data unless the exact purposes of such a request are clearly provided. Power analyses for study data that have already been analyzed have been shown to be conceptually flawed, and the interpretation of the results of such an analysis can be misleading [23,24]. In particular, using a simulation study, Zhang et al. [25] showed that post hoc power analyses for retrospective studies do not provide sensible information in terms of quantifying power to detect statistically significant differences already observed.

### 2.2. Study Design and Patients

In this retrospective study, we included all patients who underwent RM between 1 January 2019, and 28 February 2021, at Seoul Asan Hospital. Among 450 patients who underwent RM during the study period, we included only those who underwent multiport RM and excluded those who underwent single-incision RM (n = 47), single-port RM (n = 13), and single-site RM (n = 34) to minimize confounding factors related to baseline differences. We excluded one case of RM performed for parasitic myomas after total hysterectomy because the RM procedures are largely different from those performed for other types of myoma. We also excluded one case of RM that was converted to laparotomy owing to severe adhesions and more myomas than preoperatively expected. To minimize the potential impact of the surgeon factor, only 242 patients who underwent RM performed by two experienced surgeons (who each performed >80 cases of multiport RM during the study period) were included in this analysis. To further reduce confounding by the surgeon effect, the surgeon factor was adjusted for in the multivariate analysis. All included multiport RM cases were performed with a reduced port technique. “Reduced port” refers to multiport RM with a total of three skin incisions (n = 238) or 1 + 1 port with a total of two skin incisions (n = 4).

We obtained the following data from each patient’s medical chart: age, gravidity, parity, body mass index (BMI), and history of previous abdominal surgery including cesarean section. We also obtained the following surgery-related data: maximal diameter and type of myomas, number and weight of removed myomas, concomitant surgery, total OT from skin incision to closure, EBL, and blood transfusion. Data on perioperative outcomes, length of hospital stay, postoperative fever within 48 h, and any complications related to RM were also collected. Myomas were categorized by the International Federation of Gynecology and Obstetrics (FIGO) system into type 0–2 (submucosal), 3–4 (intramural), 5–7 (subserosal), or 8 (intraligamentary, cervical, and parasitic) myomas [26]. The diameter of the dominant myoma was measured on preoperative ultrasound images as Frascà et al.’s report on the role of preoperative pelvic ultrasound evaluation for LM [27]. Magnetic resonance imaging examination was performed in 152 cases (62.8%) in which there was a need to identify adenomyoma or malignancy or to ensure a more accurate assessment in cases with multiple myomas or large myomas adjacent to the uterine endometrium.

We have collected additional data on preoperative use of gonadotropin-releasing hormone agonists (GnRHas) and perioperative use of hemostatic agents (tranexamic acid or vasopressin).

This study was approved by the Asan Medical Center Institutional Review Board (approval no. 2021-0001). Informed consent was waived by our Institutional Review Board because of the retrospective chart review study design.

### 2.3. Surgical Procedures

Multiport RM was performed using the da Vinci Si or Xi system with central or side docking. Under general anesthesia, a 2.5-cm intraumbilical incision was made and a multichannel single glove port (Glove port; Nelis Meditech Inframed, Ojeong-gu, Bucheon City, Korea) was inserted. For reduced port, we made one intraumbilical incision for a multichannel single glove port for a camera and an assistant port, as well as two additional incisions on the left and right sides of the umbilical incision for 8-mm robotic trocars. For 1 + 1 port (i.e., a total of two skin incisions), we made one intraumbilical incision for a multichannel single glove port for a camera, an assistant port, and a robotic trocar plus one additional 8-mm skin incision on the left or right side of the umbilical incision for a robotic trocar.

For reduced or 1 + 1 port robotic surgery, one or two 8-mm skin incisions were made on the left and/or right sides of the umbilical incision for robotic trocars. Robotic instruments, monopolar scissors for the right robotic arm and fenestrated bipolar forceps for the left robotic arm were used for the uterine incision procedure. The right robotic arm was replaced with a Mega Suture Needle Driver^®^ during the closure of the uterine incision (i.e., during myometrial suturing). The uterine incision was closed with a two- or three-layer closure using 1-0 Monofix PDO (Samyang, Daejeon, Korea) or 1-0 V-Loc (Covidien, Mansfield, MA, USA) with a continuous running suture technique. The serosal layer was sutured with a baseball or interlocking suture technique using 1-0 V-Loc (Covidien) or 1-0 Quill™ SRS bidirectional barbed suture (Angiotech Pharmaceuticals Inc., Vancouver, BC, Canada). The retrieved myomas were extracted by manual morcellation using a scalpel, mostly within an Endobag (Sejong Medical, Paju, Gyeonggi-do, Korea). In some cases in which the myomas were too large for placement within an extra-large Endobag, the retrieved myomas were extracted by manual scalpel morcellation in an uncontained state through the existing umbilical incision. After the removal of myomas, we thoroughly examined the pelvic cavity without changing the patient’s position to check for spillage of myoma tissue. No additional skin incisions for tissue extraction were made in all cases, and all procedures were performed using robotic surgical systems without the hybrid RM technique [28]. All patients received standard postoperative care.

Normal saline, which was used for irrigation, and blood from the operative field were suctioned out into a suction bottle through a closed surgical drain system. Calculations of intraoperative fluid input and output (I/O) were performed routinely by the anesthesiologist, and the EBL was recorded immediately after the operation.

### 2.4. Statistical Analysis

Continuous data are presented as mean ± standard deviation or median with interquartile range (IQR), and between-group comparisons were performed using Student’s *t*-test or Wilcoxon rank-sum test. Categorical data are presented as numbers with percentages and were compared using the chi-square test or Fisher’s exact test, as appropriate. A two-tailed *p*-value of <0.05 was considered to indicate statistical significance. Univariate and multivariate logistic regression analyses were performed to identify independent factors associated with EBL > 320 mL and total OT > 3 h during RM. Clinical factors with *p* < 0.05 in univariate analyses or those known to be important from the literature were included in multivariate analyses. All statistical analyses were performed using R statistical software (version 3.6.3; R Foundation for Statistical Computing, Vienna, Austria, date: 19 March 2021) [29].

## 3. Results

### 3.1. Patient Baseline Characteristics

In total, 242 patients who underwent multiport RM were included in this study. The mean age and BMI were 38.01 ± 5.65 years and 22.68 ± 3.61 kg/m^2^, respectively. Of the patients, 192 (79.3%) were nulliparous women. Of the 50 parous women, 15 (30.0%) had a history of caesarian section. Most patients (227 patients, 93.8%) had no history of previous abdominal surgery, whereas 32 patients (13.2%) had a history of previous abdominal surgery. The median number of myomas was 2.00 (IQR, 1.00 to 4.00), and 76.4%, 17.8%, and 5.8% of the patients had less than five, five to nine, and more than nine myomas, respectively. The median maximal diameter of the dominant myoma and the weight of the removed myomas were 9.00 cm (IQR, 7.00 to 10.00 cm) and 249.75 g (IQR, 142.88 to 401.00 g), respectively. Data on the weight of the removed myomas were available in 194 cases. The most common type of dominant myoma was intramural (179 cases, 74.0%), followed by the subserosal (43 cases, 17.8%), intraligamentary (10 cases, 4.1%), submucosal (eight cases, 3.3%), and cervical (two cases, 0.8%) types. In logistic regression analyses, associations with the myoma type were evaluated as categories classified by intramural, subserosal, and others, and no statistical significance was found. Fourteen patients (5.8%) used a GnRHa before surgery, and tranexamic acid or vasopressin was administered to 24% or 6.2% of patients, respectively, to reduce blood loss during surgery. The mean OT was 155.29 ± 63.51 min, and the mean EBL was 384.33 ± 659.86 mL. The median length of hospital stay was two days, and the conversion rate was 0.004% (1 of 242 cases). Conversion to laparotomy was needed in one patient who was short in stature (height, 146 cm) and had multiple (>10) and large (>8 cm) myomas, which precluded successful RM because the range of motion was severely limited with the usual reduced-port method with intraumbilical incision (Table 1).

### 3.2. Multivariate Logistic Regression Analyses of EBL

The median EBL was 300 mL (IQR, 200–400 mL). We classified the patients into two groups: the high EBL group (EBL > 320 mL, 87 patients) and the low EBL group (EBL ≤ 320 mL, 155 patients). In the univariate analysis, patient age, BMI, gravidity, parity, and history of cesarean section, and medical agents such as GnRHas and tranexamic acid or vasopressin were not significantly associated with the occurrence of high EBL (*p* > 0.05).

In the multivariate analysis, the associations of EBL with a history of abdominal surgery, myoma number, maximal diameter of the dominant myoma, myoma type, and surgeon factor were assessed while adjusting for age and BMI. The odds of EBL > 320 mL were 2.51 times higher (odds ratio [OR], 2.51; 95% confidence interval [CI], 1.16–5.42; *p* = 0.019) for five to nine myomas and 6.47 times higher (OR, 6.47; 95% CI, 1.87–22.33; *p* = 0.003) for >10 myomas than for less than five myomas. In addition, the odds of having an EBL > 320 mL increased by 28% (OR, 1.28; 95% CI 1.14–1.44; *p* < 0.001) each 1-cm increase in the maximum diameter of the myoma.

The odds of EBL > 320 mL increased by 4.9 times (OR, 4.90; 95% CI, 2.02–11.92; *p* < 0.001) in the presence of a history of abdominal surgery.

The odds of EBL > 320 mL were lower by 67% (OR, 0.33; 95% CI, 0.14–0.80; *p* = 0.014) in the subserosal-type than in intramural-type myomas, whereas the odds for other myoma types were not significant (Table 2). The OR for the surgeon factor was not significantly different from 1 (*p* = 0.55), and medical agents (*p* > 0.05) were not significantly different.

### 3.3. Multivariate Logistic Regression Analyses of Total OT

The median OT was 144 min (IQR, 113–182 min). We classified the cases into two groups: total OT > 3 h (62 cases) and total OT ≤ 3 h (180 cases). The univariate analysis results showed that parity, gravidity, and medical agents such as GnRHas and tranexamic acid or vasopressin were not significantly associated with the binary operative outcomes. The multivariate analysis results showed that patient’s age, BMI, history of cesarean section, history of abdominal surgery, maximal diameter of the dominant myoma, and myoma types did not significantly differ between the total OT > 3 h group and the total OT ≤ 3 h group (*p* > 0.05). However, the associations with myoma number and surgeon factor remained significant even after adjusting for other factors in Table 3. The odds of total OT > 3 h were 2.72 times higher (OR, 2.72; 95% CI, 1.22–6.07; *p* = 0.014) for five to nine myomas and 16.13 times higher (OR, 16.13; 95% CI, 4.36–59.71; *p* < 0.001) for >10 myomas (Table 3) than for less than five myomas.

## 4. Discussion

In this study, we identified the factors affecting EBL and total OT in multiport RM. The number of myomas was positively correlated with both EBL and total OT; however, the maximal myoma diameter was positively correlated with only EBL and was not related to total OT. The subserosal type of dominant myoma was negatively correlated with EBL but was not related to total OT. The surgeon factor was related to total OT but not to EBL.

On the basis of the study results, higher EBL and longer total OT can be expected in RM for more than five myomas, especially more than 10 myomas. Contrary to previous reports [30,31,32], the maximal myoma diameter affected only the EBL and did not affect the total OT of minimally invasive myomectomy in our study. Therefore, the number of myomas, especially if more than 10, should be considered the main limitation to the indications for performing RM, even in multiport RM. Although we did not include single-incision RM in this study, our results can also be applied to single-incision RM, as multiport RM is usually easier to perform than single-incision RM.

### 4.1. Previous Reports on EBL and Total OT in RM

In total, RM was first described by Advincula et al. in 2004 in 35 cases with a mean myoma weight of 223.2 ± 244.1 g and a mean number of removed myomas of 1.6. The mean OT was 230.8 ± 83 min and the mean EBL was 169 ± 198.7 mL [30]. In 2007, the first study comparing RM and abdominal myomectomy (AM) was published. The study reported decreased EBL, complication rates, and the length of stay with RM but identified the high cost of RM as the main disadvantage of this procedure [33]. In 2013, a meta-analysis comparing RM with either LM or AM was published. The study reported that RM was associated with less EBL and a lower risk of blood transfusion than AM; however, no significant difference was found between RM and LM [34]. A recent study comparing RM (n = 126) and AM (n = 151) for large myomas (>10 cm or 250 g) observed no difference in EBL between the two groups [35]. A recent meta-analysis including a total of 2027 patients found no significant difference between RM and LM in terms of OT, EBL, blood transfusion, length of hospital stay, complications, and postoperative fertility [21]. Minimally invasive techniques were reported to have a longer OT [16], and AM is often recommended for large or multiple myomas. Long OT was the main disadvantage of RM, especially in the early period of the use of this technique [13,15].

### 4.2. Our Opinion on the Innate Limited Advantages of RM Compared with LM in Terms of EBL

In a meta-analysis design, the difference in blood loss between LM and RALM was only 42 mL, which was not statistically significant and is certainly not clinically significant [17]. Although articles comparing LM and RM did not report significant differences in EBL [17,36], RM can have limited advantages during the myoma retrieval and hemostasis procedures compared with LM [37], and we believe that this can affect the total OT and EBL. The limited availability of instruments for hemostasis during RM is one of the reasons. Fenestrated bipolar forceps, Maryland bipolar forceps, monopolar scissors, and a monopolar hook bovie are the available robotic instruments for RM. Although all these instruments can effectively perform hemostasis, they take a longer time to achieve hemostasis than the highly efficient, newly developed, and diverse advanced energy devices for laparoscopy. Although Vessel Sealer^®^ (Intuitive Surgical, Sunnyvale, CA, USA) was introduced for use in robotic surgical systems in 2018, the working time remains long, the hemostatic effect is not considerably strong, and it is not as easy to use as the diverse energy devices for laparoscopy, from our experience.

Another reason for the possibly limited advantage of RM in terms of hemostasis compared with LM is that during robotic surgery, extra time is needed for changing robotic instruments. That is, the previously used instrument needs to be dismantled and another robotic instrument needs to be installed for use. The robotic system needs to recognize the installed instruments, which is not necessary during laparoscopic surgery. Although the time gap is short (only approximately 5 s), achieving hemostasis in the shortest time possible is desirable.

Furthermore, robotic instruments have a high cost, especially Vessel Sealer^®^, which is approximately 1.5 times more expensive than laparoscopic energy devices, thus making surgeons reluctant to use it. Moreover, only 8-mm robotic devices are available for RM, and 5- to 6-mm devices are available only for single-incision robotic systems. In contrast, laparoscopic energy devices are available in sizes of 10 and 5 mm.

A hybrid technique has been developed to overcome the above-mentioned limited advantages of RM compared with LM [28]. The hybrid technique consists of the initial surgical steps of myomectomy, but uterine incision and myoma retrieval are performed using conventional laparoscopy, which enables the use of effective energy devices for hemostasis and laparoscopic tenaculum forceps or myoma screw for myoma traction. The subsequent surgical step, multiple intracorporeal myometrial suturing, is performed with robotic surgical systems using the effective EndoWrist^®^ technology. The authors reported that taking advantage of both surgical approaches resulted in the reduction of the OT [30]. However, it is rather cumbersome to prepare both surgical systems (laparoscopic and robotic systems) for hybrid minimally invasive surgery in all cases of RM. Moreover, we used barbed suture materials, which have been proven to be effective in reducing the OT and EBL and are associated with similar pregnancy outcomes to those achieved when conventional suture materials have been used [38,39]. Therefore, knowing the factors associated with blood loss during RM will be helpful in the preoperative determination of whether RM needs to be performed with the hybrid technique or other methods proven to decrease the EBL, such as barbed suture materials. It will also be useful in determining the need for preparing blood or plasma replacement products, using all kinds of preoperative and intraoperative methods proven to decrease intraoperative EBL, and advising the patient to take iron-containing products preoperatively, even if the preoperative hemoglobin level is within the normal limit, to decrease the risk of intraoperative and postoperative transfusion for large amounts of blood loss.

### 4.3. Strengths and Limitations

This study has several strengths. First, there was a minimal difference in surgical proficiency between the two surgeons who each performed >80 RM procedures during the study period. Second, the relatively short study period of 26 months in the present study can minimize the intrapersonal variations in the surgical experience. Third, none of the cases required an additional skin incision, and only one case needed conversion to mini-laparotomy. Fourth, we provided data on the preoperative use of GnRHas and perioperative use of medical agents to decrease blood loss, such as tranexamic acid and vasopressin. Most of these factors could not be adjusted for in previous retrospective studies. As a sensitivity analysis, we explored the effects of these medical agents in univariate and multivariate analyses, and we found that those medical agents’ use was not associated with having high EBL and longer OT (*p* > 0.05).

Nevertheless, this study also had some limitations. First, this was not a multicenter study, thus precluding the universal application of the study results to all RM procedures. Second, the impact of the different da Vinci systems (Si, Xi, and SP) were not applied as statistical analysis; however, according to the two surgeons’ opinion, who performed all these cases and who have used all kinds of the da Vinci system (Si, Xi, and SP), the different da Vinci systems cannot affect the EBL. Third, we could not evaluate fertility outcomes. The only fertility data that we can obtain from this retrospective chart review was on three patients who are on follow-up at our institute. Three patients became pregnant during the study period and two of them gave birth at full term through Caesarean section. The other patient became pregnant and is now 30 weeks pregnancy. There was no case of uterine rupture during pregnancy.

## 5. Conclusions

The number of myomas (five to nine or ≥10) affects both EBL > 320 mL and total OT > 3 h, and the maximal myoma diameter and history of abdominal surgery other than cesarean section affect the EBL but not the total OT in multiport RM.

## Figures and Tables

**Table 1:** Baseline characteristics of patients.

Factors		Total (n = 242)	EBL ≤ 320 mL (n = 155)	EBL > 320 mL (n = 87)	*p*-Value	OT ≤ 3 h (n = 180)	OT > 3 h (n = 62)	*p*-Value
Age (years, mean ± SD)		38.01 ± 5.66	37.57 ± 5.73	38.80 ± 5.47	0.103	37.88 ± 5.59	38.40 ± 5.89	0.529
BMI (kg/m^2^, mean ± SD)		22.68 ± 3.62	22.61 ± 3.68	22.82 ± 3.52	0.665	22.61 ± 3.68	22.89 ± 3.46	0.598
Parity (%)	0	192 (79.3%)	120 (77.4%)	72 (82.8%)	0.325	139 (77.2%)	53 (85.5%)	0.166
	≥1	50 (20.7%)	35 (22.6%)	15 (17.2%)		41 (22.8%)	9 (14.5%)	
Gravidity (%)	0	166 (68.6%)	103 (66.5%)	63 (72.4%)	0.338	123 (68.3%)	43 (69.4%)	0.881
	≥1	76 (31.4%)	52 (33.5%)	24 (27.6%)		57 (31.7%)	19 (30.6%)	
Previous cesarean section	No	227 (93.8%)	145 (93.5%)	82 (94.3%)	0.827	167 (92.8%)	60 (96.8%)	0.366
	Yes	15 (6.2%)	10 (6.5%)	5 (5.7%)		13 (7.2%)	2 (3.2%)	
Previous pelvic surgery	No	210 (86.8%)	143 (92.3%)	67 (77.0%)	0.001	157 (87.2%)	53 (85.5%)	0.727
	Yes	32 (13.2%)	12 (7.7%)	20 (23.0%)		23 (12.8%)	9 (14.5%)	
No. of myomas (median [IQR])		2.00 [1.00–4.00]	2.00 [1.00–3.50]	3.00 [1.00–5.50]	0.002	2.00 [1.00–3.25]	4.00 [2.25–7.75]	<0.001
No. of myomas (%)	1–4	185 (76.4%)	129 (83.2%)	56 (64.4%)	0.003	148 (82.2%)	37 (59.7%)	<0.001
	5–9	43 (17.8%)	21 (13.5%)	22 (25.3%)		28 (15.6%)	15 (24.2%)	
	≥10	14 (5.8%)	5 (3.2%)	9 (10.3%)		4 (2.2%)	10 (16.1%)	
Maximal myoma diameter (median [IQR])		9.00 [7.00–10.00]	9.00 [7.00–10.00]	10.00 [8.00–12.00]	0.001	9.00 [7.00–10.00]	9.00 [8.00–10.00]	0.126
No. of myomas with Maximal diameter ≥ 3 cm		1.00 [1.00–2.00]	1.00 [1.00–2.00]	2.00 [1.00–3.00]	0.06	1.00 [1.00–2.00]	2.00 [1.00–4.00]	<0.001
Weight of removed myomas (g, median [IQR]) *		249.75 [142.88–401.00]	225.50 [132.88–347.00]	369.50 [189.00–543.50]	<0.001	235.00 [140.00–370.00]	346.00 [168.75–579.00]	0.017
Type of main myoma	Intramural (FIGO 3–4)	179 (74.0%)	110 (71.0%)	69 (79.3%)	0.630	134 (74.4%)	45 (72.6%)	0.614
	Subserosal (FIGO 5–7)	43 (17.8%)	31 (20.0%)	12 (13.8%)		29 (16.1%)	14 (22.6%)	
	Others (FIGO 0–2 and 8)	20 (8.2%)	14 (9.0%)	6 (6.9%)		17 (9.4%)	3 (4.8%)	
Operator (%)	K.S.H L.S.R	83 (34.3%) 159 (65.7%)	61 (39.4%) 94 (60.6%)	22 (25.3%) 65 (74.7%)	0.027	57 (31.7%) 123 (68.3%)	26 (41.9%) 36 (58.1%)	0.142
Medical agents (%)	GnRHa	14 (5.8%)	11 (7.1%)	3 (3.4%)	0.39	10 (5.6%)	4 (6.5%)	0.759
	Tranexamic acid	58 (24%)	33 (21.3%)	25 (28.7%)	0.193	45 (25%)	13 (21%)	0.521
	Vasopressin	15 (6.2%)	9 (5.8%)	6 (6.9%)	0.784	8 (4.4%)	7 (11.3%)	0.068

* n = 194. SD, standard deviation; EBL, estimated blood loss; OT, total operation time from skin incision to skin closure; IQR, interquartile range; BMI, body mass index; FIGO, International Federation of Gynecology and Obstetrics; GnRHa, gonadotropin-releasing hormone agonist.

**Table 2:** Univariate and multivariate logistic regression analyses of EBL > 320 mL.

Factors		Univariate OR (95% CI) *p*-Value		Multivariate OR (95% CI) *p*-Value	
Age		1.04 (0.99–1.09)	0.104	1.02 (0.97–1.08)	0.47
BMI		1.02 (0.94–1.09)	0.663	1.00 (0.92–1.08)	0.973
Parity	0 ≥1	Reference 0.71 (0.36–1.40)	0.326		
Gravidity	0 ≥1	Reference 0.76 (0.42–1.34)	0.338		
Previous cesarean section	No Yes	Reference 0.88 (0.29–2.67)	0.827		
Previous pelvic surgery	No Yes	Reference 3.56 (1.64–7.70)	0.001	Reference 4.90 (2.02–11.92)	<0.001
No. of myomas	1–4 5–9 ≥10	Reference 2.41 (1.23–4.74) 4.15 (1.33–12.93)	0.011 0.014	Reference 2.51 (1.16–5.42) 6.47 (1.87–22.33)	0.019 0.003
Maximal myoma diameter		1.21 (1.10–1.34)	<0.001	1.28 (1.14–1.44)	<0.001
Type of main myoma *	Intramural (FIGO 3–4) Subserosal (FIGO 5–7) Others (FIGO 0–2 and 8)	Reference 0.62 (0.30–1.28) 0.68 (0.25–1.86)	0.196 0.456	Reference 0.33 (0.14–0.80) 0.83 (0.26–2.63)	0.014 0.752
Operator (%)	K.S.H L.S.R	Reference 1.92 (1.07–3.43)	0.028	Reference 1.23 (0.62–2.43)	0.55
GnRHas	No Yes	Reference 0.47 (0.13–1.72)	0.253	Reference 0.59 (0.13–2.60)	0.482
Tranexamic acid or vasopressin	No Yes	Reference 1.49 (0.85–2.62)	0.166	Reference 1.27 (0.66–2.45)	0.465

EBL, estimated blood loss; OR, odds ratio; Cl, confidence interval; BMI, body mass index; Ref, reference; FIGO, International Federation of Gynecology and Obstetrics; GnRHas, gonadotropin-releasing hormone agonists.

**Table 3:** Univariate and multivariate logistic regression analyses of operation time > 3 h.

Factors		Univariate OR (95% CI) *p*-Value		Multivariate OR (95% CI) *p*-Value	
Age		1.02 (0.97–1.07)	0.528	1.02 (0.96–1.08)	0.552
BMI		1.02 (0.94–1.10)	0.596	1.03 (0.94–1.12)	0.508
Parity	0 ≥1	Reference 0.58 (0.26–1.27)	0.170		
Gravidity	0 ≥1	Reference 0.95 (0.51–1.78)	0.881		
Previous cesarean section	No Yes	Reference 0.43 (0.09–1.95)	0.273	Reference 0.46 (0.09–2.31)	0.342
Previous pelvic surgery	No Yes	Reference 1.16 (0.50–2.66)	0.728	Reference 1.38 (0.56–3.40)	0.488
No. of myomas	1–4 5–9 ≥10	Reference 2.14 (1.04–4.42) 10.00 (2.97–33.68)	0.039 <0.001	Reference 2.72 (1.22–6.07) 16.13 (4.36–59.71)	0.014 <0.001
Maximal myoma diameter		1.05 (0.95–1.16)	0.290	1.10 (0.98–1.23)	0.119
Type of main myoma *	Intramural (FIGO 3–4) Subserosal (FIGO 5–7) Others (FIGO 0–2 and 8)	Reference 1.44 (0.70–2.96) 0.52 (0.15–1.88)	0.324 0.322	Reference 1.09 (0.49–2.42) 0.81 (0.21–3.05)	0.838 0.756
Operator (%)	K.S.H L.S.R	Reference 0.64 (0.35–1.16)	0.143	Reference 0.34 (0.16–0.70)	0.004
GnRHas	No Yes	Reference 1.17 (0.35–3.88)	0.795		
Tranexamic acid or vasopressin	No Yes	Reference 1.14 (0.61–2.12)	0.677		

OR, odds ratio; Cl, confidence interval; BMI, body mass index; Ref, reference; FIGO, International Federation of Gynecology and Obstetrics; GnRHa, gonadotropin-releasing hormone agonists.

## Data Availability

The excel data used to support the findings of this study were supplied by Sa Ra Lee under license, and requests for access to these data should be made to S.R.L. leesr@amc.seoul.kr.
